# PLGA nanoparticles for capsaicin delivery: enhanced encapsulation efficiency and pro-apoptotic activity in HEPG2 cells

**DOI:** 10.3389/fbioe.2025.1617022

**Published:** 2025-07-18

**Authors:** Chiara Mulè, Tania Mariastella Caputo, Antonio Montefusco, Antonio Massimiliano Romanelli, Ivana Caputo, Gaetana Paolella, Anna Aliberti, Andrea Cusano

**Affiliations:** ^1^ Optoelectronics Group, Department of Engineering, University of Sannio, Benevento, Italy; ^2^ Department of Chemistry and Biology, University of Salerno, Fisciano, Italy; ^3^ CeRICTscrl Regional Center Information Communication Technology, Benevento, Italy

**Keywords:** capsaicin, PLGA nanoparticles, HepG2, apoptosis, cap encapsulation efficiency, drug delivery

## Abstract

**Introduction:**

Capsaicin (trans-8-methyl-N-vanillyl-6-nonenamide) (Cap) is a lipophilic alkaloid derived from Capsicum annuum. It was observed that Cap has an antitumoral activity in several cancer types, in particular in liver, colon and breast cancer. Actually, the use of Cap in the cancer therapy is limited by its very low bioavailability, a short half-life and side effects as mouth and stomach irritations and burning sensation. To overcome these limitations, the Cap has been encapsulated in carriers in order to reduce the adverse effect and to help the delivery in the cancer cells. In this study, we synthesized Poly(lactic co-glycolic acid) (PLGA) nanoparticles (NPs) to encapsulate Cap (PLGA-Cap), optimizing the synthetic strategy and improving its efficiency and safety. This is the first time that PLGA-Cap NPs was tested on HepG2 cells line for Hepatocellular carcinoma (HCC) therapy.

**Methods:**

NPs are characterized by Dynamic Light Scattering (DLS), Fourier transform infrared spectroscopy (FTIR), Morphological analysis by scanning transmission electron microscopy (STEM) and Reverse-Phase High Liquid Chromatography (RP-HPLC) to study their physicochemical properties and the best condition in terms of size, PDI and encapsulation efficiency. In vitro biological MTT assay was performed on HepG2 cells to observe the cell proliferation in response to PLGA-Cap. The apoptosis induced by Cap was evaluated the enzymatic activity of caspase 3, Bcl2 and Bax expression by Western blot and ROS activity.

**Results and Discussion:**

Our preparation showed the highest Encapsulation Efficiency (96%) reported by the literature, showing an improvement of 21% compared to what is actually reported. In vitro experiments revealed that PLGA-Cap formulation induced similar biological effects in terms of cell viability compared to free Cap. Moreover, HepG2 cancer cells treated with PLGA-Cap exhibited increased caspase 3 activity respect to those treated with free Cap.

**Conclusion:**

In conclusion we demonstrated that our preparation showed an improvement in encapsulation parameters and in pro-apoptotic and anticancer activity in HepG2 cells.

## 1 Introduction

Capsaicin (*trans*-8-methyl-N-vanillyl-6-nonenamide) (Cap) is a lipophilic alkaloid derived from *Capsicum annuum* that is responsible for pungent sensation and hot flavour of chili peppers (Al-Samydai et al., 2021). It has several health benefits, including anti-inflammatory, antioxidant, antimicrobial, cardio-protective, gastro-protective, anti-obesity and analgesic effect. Over time, Cap has been shown to exhibit anti-cancer effects on more than 40 cancer cell lines, including pancreas, colon, prostate, liver, bladder, lung types (Arul and Kothai, 2020) Cap is a high affinity agonist of the transient receptor potential vanilloid (TRPV1) receptor (Andresen, 2019; Li L. et al., 2021). However, there are several data suggesting that the biological functions of capsaicin may be mediated via TRPV1-dependent or TRPV1-independent pathways. (Arul and Kothai, 2020; Zhang et al., 2020). Capsaicin (Cap) acts on TRPV1, a non-selective ion channel responsible for regulating intracellular calcium. Amantini et al. (2007) demonstrated that Cap induces apoptosis in U373 glioma cells by elevating calcium levels, promoting phosphatidylserine exposure, disrupting mitochondrial membrane potential, and causing DNA fragmentation—all of which were prevented by the TRPV1 antagonist capsazepine (Amantini et al., 2007). In prostate cancer cell lines Cap reduce proliferation by triggering the TRPV1 and downstream axis LKB1/AMPK (Sánchez, B.G et al., 2022); furthermore, Cap is active also in metastasis process reducing migration and invasion in papillary thyroid cancer BCPAP cells by TRPV1 activation (Xu S et al., 2018). Furthermore, Cap triggers apoptosis through the generation of reactive oxygen species (ROS). In pancreatic cancer cells, N-acetylcysteine effectively reduced Cap-induced ROS production and mitochondrial dysfunction, a finding corroborated by preclinical studies using xenograft mouse models (Zhang et al., 2008). More studies conducted on Hepatocellular carcinoma cell line have shown that treatment with Cap increased the production of ROS and induced the overexpression of HO-1, an important detoxifying/antioxidant enzyme (Joung et al., 2007), through a sequence of Cap-induced events. Cap has been shown to induce apoptosis and mitochondrial dysfunction through ROS generation in various *in vitro* models, including colon, brain, and liver tumor cells (Yang et al., 2024; Chen et al., 2016; Huang et al., 2009). In HepG2 tumor cells and in normal hepatocytes (HL-7702), Cap triggered oxidative stress by reducing antioxidative factors and led to apoptosis via the SIRT1/NOX4 signaling pathway (Hacioglu, 2022). Likewise, in melanoma cells, Cap was shown to inhibit the SIRT/tNOX axis, thereby promoting ROS-dependent autophagy, as evidenced by both *in vitro* and *in vivo* studies (Islam A et al., 2021). Furthermore, Cap induced an increase in intracellular Ca^2+^, which reduced mitochondrial membrane potential, leading to the release of cytochrome C from the mitochondria into the cytosol. Subsequently, caspase-9 and -3 were activated leading to cell death by apoptosis (Huang et al., 2009).

Specifically, on HepG2 cells, the study by Huang et al. (2009) highlighted that Cap induced increased expression of GRP78 and GADD153, markers of endoplasmic reticulum stress, mitochondrial dysfunction with loss of membrane potential and alteration of the protein family implicated in apoptosis. In particular, it was recorded that the pro-apoptotic protein Bax increased its expression and that the anti-apoptotic protein Bcl-2 reduced its expression in the mitochondrial fraction. Instead, p53 and cytochrome c increased their cytosolic expression following exposure to Cap (Huang et al., 2009). This influence of Cap on proteins involved in apoptosis has also been documented in other cell lines such as SK-Hep-1 where Cap induced a reduction in the ratio between Bcl-2 and Bax with consequent activation of caspase-3 and DNA fragmentation (Jung et al., 2001).

In human gastric carcinoma cells, treatment with Cap leads to an upregulation of the expression of phosphorylated ERK 1/2, P38 MAPK, or JNK driving the tumour cells to apoptotic death (Park et al., 2014). The anti-proliferative effect of Cap was also demonstrated in prostate stem cells (CSC). The molecule was shown to suppress CSC markers and the growth of PC-3 and DU145 prostate CSCs by regulating the expression of Wnt-2, via the Wnt/β-catenin pathway (Zhu et al., 2020; Chen et al., 2021). The use of natural compounds in combination with chemotherapy drugs is a possible strategy to increase the anti-tumor response. Synergistic anticancer effects are observed when Cap is combined with established chemotherapeutic agents. Cap enhances camptothecin-induced apoptosis in small cell lung cancer via increased intracellular Ca^2+^ and calpain activity. In hepatocellular carcinoma, Cap potentiates sorafenib’s antiproliferative and pro-apoptotic effects (Zhang et al., 2018). Similarly, Cap enhances cisplatin’s antiproliferative, antimigratory, and pro-apoptotic activity in osteosarcoma cells, also reducing tumor growth in xenograft models (Wang et al., 2018). Furthermore, Cap mitigates doxorubicin-induced cardiotoxicity by modulating the PI3K/Akt signaling pathway and iron homeostasis (Wang et al., 2023). These studies highlight Cap’s potential to improve cancer therapy efficacy and reduce chemotherapy-related side effects (Wang et al., 2023). Unfortunately, the use of Cap as an anticancer agent still has some drawbacks, that limit the use of Cap in clinical practice. First of all, Cap has very low bioavailability due to its hydrophobic nature and a short half-life as it is easily metabolised by the liver. In addition, oral administration of Cap can cause mouth and stomach irritations (Srinivasan, 2016), while, intravenous administration can cause a burning sensation due to the higher dose required to achieve the cytotoxic effect (Rollyson et al., 2014). To overcome these dramatic limitations and maintain the benefits of Cap, new delivery methods need to be developed.

A promising strategy to improve the stability and solubility of the Cap focuses on the development of suitable nanocarriers that can encapsulate the Cap and promote its transport through biological membranes, thus prolonging its circulation time and increasing its safety and efficacy. To this purpose, a new focus has emerged in recent years on the development of different Cap-loaded nanocarriers. For example, biodegradable polymeric carriers (MPEG-PCL-NPs) with a diameter of 82.54 ± 0.51 nm and encapsulation efficiency (EE) of 81.5% were tested in rats, and a reduction of gastric mucosa irritation was observed (Peng et al., 2015). Abdelnabi et al., synthesized Cap-complexed β-cyclodextrin pegylated liposomes (181 ± 36 nm, EE 38.65%) capable to induce a significant reduction in IL-8 production in MDA-MB-231 (Triple-Negative Breast cancer) and A549 (Non-small cell lung cancer) cells (Abdelnabi et al., 2021). Similarly, nanomicelles with a size of 29.9 ± 0.8 nm obtained by self-assembly of α-lactalbumin polypeptides exhibited a considerable potential for delivery of Cap in the steatotic HepG2 cell model, suggesting that encapsulation can enhance the molecule penetration into cells (Zuo et al., 2021). Among polymers, Poly (lactic co-glycolic acid) (PLGA) is a Food and Drug Administration (FDA)-approved biodegradable polymer widely used in cancer nanotechnology. PLGA is an appealing material for nanocarrier development because it is biocompatible and biodegradable and can encapsulate molecules of various nature and dimension, both hydrophilic and hydrophobic (Loureiro and Pereira, 2020; Caputo et al., 2023b; 2023a). Recently, a sophisticated chemo-photodynamic therapy based on PLGA nanosystems has been developed for the treatment of triple-negative breast cancer (TNBC). First, a hybrid membrane (HM) camouflaged PLGA nanosystem was developed for the delivery of Cap to the tumour (HMPLGA@Cap NPs). PLGA was then also co-loaded with Gamabufotalin (CS-6) and photosensitizer Chlorin e6 (Ce6), which penetrate into tumour core regions. This sequential delivery strategy inhibited both tumour growth and metastasis of primary TNBC (Fan et al., 2022). The most relevant paper with these results are reported in the Table 5.

To our knowledge, there is currently no evidence in the literature of biocompatible polymeric PLGA nanoparticles used to encapsulate Cap that have been tested on HepG2 cells for the treatment of HCC (Fan et al., 2022). In this work we focus on the optimization of a synthetic strategy to encapsulate Cap with high EE within the hydrophobic core of the nanoparticles. A careful investigation of the morphological, structural, and physicochemical properties of PLGA particles has been carried out, focusing on particle size, surface charge, drug loading content, encapsulation efficiency, and *in vitro* test on HepG2 cell line.

## 2 Experimental section

### 2.1 Materials

PLGA (Resomer^®^ 504 H, 50:50 lactide: glycolide, acid terminal, MW 40.000 Da) was purchased by Sigma-Aldrich Co. Poly Vinyl Alcohol (PVA), Ethyl acetate (EtOAc), Capsaicin, trehalose, Phosphate Buffer Saline (PBS) Tween 20 and Dimethyl sulfoxide (DMSO) were purchased from Sigma-Aldrich Co. (Merk KGaA, St. Louis, MO, United States). Water, acetonitrile and trifluoroacetic acid (TFA) LC-MS grade were from (ROMIL Ltd, Cambridge, UK). Deionized water (18.2 MΩcm) was obtained from a Milli-Q system (Merck Millipore, St. Louis MO, United States).

### 2.2 Particle synthesis

Cap-loaded PLGA-nanoparticles (PLGA-Cap) were synthesized using the single emulsion solvent evaporation method. The oil phase was prepared by mixing 1 mg of cap (33 uL of the 30 mg/mL stock solution in ethyl acetate) and 5 mg of PLGA (500 μL, 10 mg/mL, dissolved in ethyl acetate) following 2 min of sonication. The oil phase was added to 5 mL of an aqueous PVA solution (0.5% w/v) and the phases were emulsified by sonication with a microtip (Sonifier™ SFX150, Branson Ultrasonics, Emerson Electric Co, St. Louis, MO, United States). Different ultrasonication protocols were tested in order to optimize the synthesis. In particular, time of activation and, percentage of amplitude were combined testing 18 possible combinations. After the ultrasonication step, the nanoparticle solution was left to stir overnight at room temperature. Finally, the nanoparticles were collected and washed twice with milli-Q water by centrifugation at 20,000 rcf and 4°C for 1 hour. Finally, the particles were freeze-dried by Alpha 1-2 LD (Christ, Memmingen, Germany) adding 1.25% w/v of trehalose as cryoprotectant. Empty PLGA particles (PLGA-N) were prepared with the same synthetic procedure. The process yield was calculated after the freeze-drying process as the ratio of the collected NPs to the starting raw materials (Cap and polymers, cryoprotectant).

### 2.3 Nanoparticle characterization by dynamic light scattering (DLS)

The hydrodynamic size of particles, the zeta potential, and Poly Dispersion Index (PDI) were measured by DLS (Malvern Zetasizer Nano ZS instrument, 633 nm laser, dispersion angle of 173°) at fixed temperature (25°C) using a material refractive index (RI) of 1.59 with a dispersant RI of 1.33. All the formulations were diluted in milli-Q water (0.3 mg/mL) and measurements were performed with an equilibration time of 120 s for a total of 5 runs.

### 2.4 Analysis by fourier transform infrared spectroscopy (FTIR)

PLGA particles (Naked and Cap-loaded) and free Cap were analysed by Fourier Transform Infrared Spectroscopy (FTIR). Spectral analysis was carried out in the Spectrum 3 spectrometer (Perkin-Elmer, Inc. Waltham, MA, USA) equipped with a total attenuated reflectance accessory (UATR). The spectra were collected by performing 10 scans with a resolution of 4 cm^−1^ in the region of 650–4,000 cm^−1^.

### 2.5 Morphological analysis by scanning transmission electron microscopy (STEM)

Images of PLGA particles (Naked and Cap-loaded) were collected by using the highly automated Thermo Scientific™ PhenomPharos™ G2 FEG-SEM with the scanning transmission electron microscopy (STEM) detector. Freeze dried particles were solubilized in water, and 5 µL of the solution was deposited on special TEM copper grids with a carbon coated Formvar. After evaporation of the solution, the grid was washed with deionized water, and loaded on the STEM sample holder.

### 2.6 Capsaicin quantification by reverse-phase high liquid chromatography (RP-HPLC)

**FIGURE 1:**
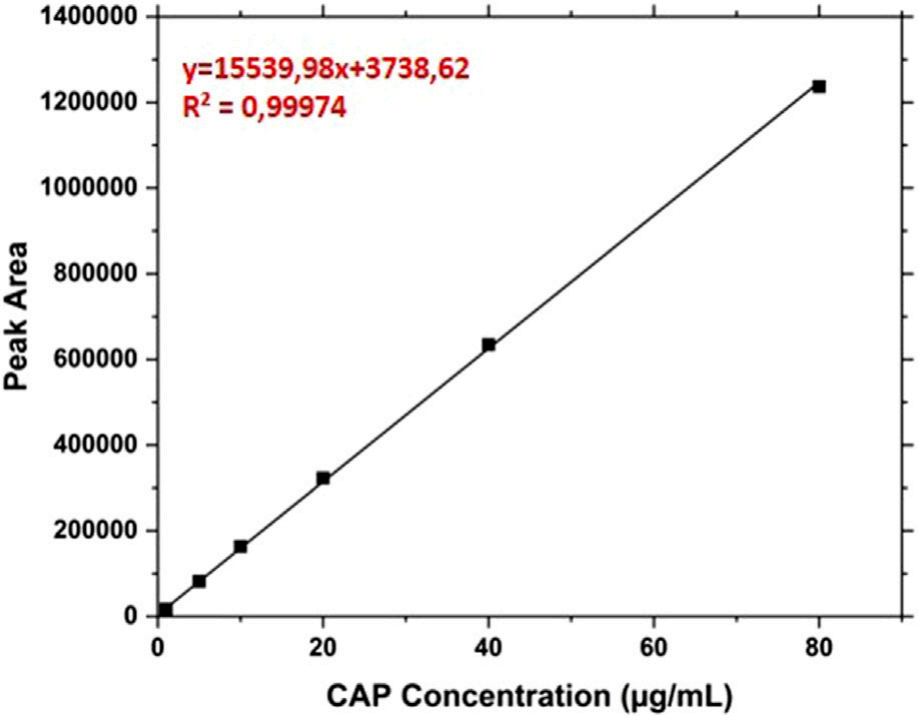
Calibration line of the free Capsaicin from 1 μg/mL to 80 μg/mL.

The RP-HPLC analysis of Cap was made by a cromatographic apparatus UltiMate 3,000 DIonex (Thermo-Fisher Scientific) equipped with a C18 BioBasic ™ column (50 × 2,1 mm, 5 μm), thermostat control at 37°C and UV detection at 227 nm. The elutions were performed at 0.2 mL/ min from the mobile phases A (trifluoroacetic acid 0.08% v/v in water) and B (trifluoroacetic acid 0.08% in acetonitrile) with the following gradient referred to the percentage of B: from 30% to 80% in 15 min; from 80% to 90% in 1 min; 90% from 16 to 21 min; from 90% to 30% in 1 min; 30% for 8 min. In this condition, the retention time of CAP was about 8.4 min. The CAP concentrations were calculated in relation to the calibration curve created by analyzing solutions between 1 and 80 μg/mL (Figure 1).

### 2.7 Encapsulation efficiency and drug loading rate

The encapsulation efficiency (EE) and the drug loading (DL) of the PLGA-NPs was assessed via RP-HPLC. Specifically, the particles were dissolved in DMSO at concentration of 5 mg/mL. The solution was centrifuged at 16,000 rcf for 15 min at room temperature, therefore, 5 µL of the supernatant were analysed as described in the previous paragraph.

The encapsulation efficiency (EE) was calculated as:

$$EE\left(\%\right)=\frac{\left(Ci-Cf\right)}{Ci}*100$$

where Ci is the theoretical initial concentration of Cap added and Cf is the free Cap concentration recovered in the supernatant after centrifugation.

The percentage of drug loaded (DL) was calculated as follows:

$$DL\left(\%\right)=\frac{\left(We\right)}{Wp}*100$$

where We is the weight of the encapsulated cap and Wp is the weight of the particles.

### 2.8 *In vitro* drug release study

The dialysis bag methodology was implemented for assessing the *in vitro* release study following prior published methodologies (Caputo et al., 2023a; 2023b). Briefly, 1 mg of PLGA-NPs loaded with CAP were added to 100 uL of release buffer (phosphate buffer pH 7.4 supplemented with Tween 20 0.3% w/v). The particle solution was loaded into a microdialysis filters (Pierce™ 96-well Microdialysis Plate, cut-off 3.5 K MWCO), and immersed in 1 mL of the release buffer. The solutions were kept in continuous agitation at 37°C, and at specific time points the external solution was fully recovered and replaced with 1 mL of fresh release buffer. The release test was run for 7 days. Finally, the particles loaded into the microfilter were collected and dissolved in DMSO to quantify the residual molecules. All the recovered aliquots were analysed using RP-HPLC. The results were expressed as mean values +/− standard deviation of three independent measurements.

### 2.9 Biological *in vitro* study

#### 2.9.1 Cells culture

To evaluate the biological activity of PLGA-Cap or Cap, a human hepatocellular carcinoma cell line (HepG2) was selected. The cell line was obtained from Interlab Cell Line Collection (IST, Istituto Nazionale per la Ricerca sul Cancro, Genoa, Italy) and was cultured in Eagle’s Minimum Essential medium (Life Technologies, Milan, Italy) supplemented with 10% (v/v) fetal bovine serum, 1% (v/v) non-essential amino acids, 0.2 mM L-glutamine, 50 units/mL penicillin and 50 μg/mL streptomycin. Cells were maintained at 37°C, in a 5% CO_2_, 95% air humidified atmosphere and passaged twice a week. For cell treatments, a stock solution of free Cap was prepared in DMSO and dilutions were done in culture medium. A stock solution of PLGA-Cap was also prepared in culture medium; in this starting solution, the calculated concentration of Cap was 0.5 mM.

#### 2.9.2 Cell viability assay

To perform the cell viability assay, HepG2 cells were plated at density of 1.6 × 10^4^/cm^2^ in 96-wells plate, cultured for 24 h and then treated with PLGA-Cap or Cap for 24 h, 48 h, and 72 h. Then, 3- (4,5- dimethylthiazol-2-yl)-2,5-diphenyl tetrazolium bromide (MTT) salt 0.5 mg/mL (Sigma- Aldrich) was added to cell and incubated for 1.5 h at 37°C; the resulting formazan crystals were dissolved in DMSO and absorbance measured at 595nm and 655 nm using a microplate reader spectrophotometer (Spectramax mini, Molecular Devices). Finally, from 595 nm signals were subtracted background signals at 655 nm and data were expressed as percentage of viability cell vs. vehicle or PLGA-N.

#### 2.9.3 Caspase 3 assay

Caspase 3 activity was analyzed using EnzChek caspase-3 assay kit (Thermo-Fisher Scientific) in according with manufacturer’s specifications. Briefly, HepG2 cells were plated in 6-wells plate at density of 3.5 × 10^4^/cm^2^ and, after 48 h, treated with PLGA-Cap or Cap for 6 h. Cells were harvested in PBS and lysed in 1X cell lysis buffer on ice for 30 min, then centrifuged at 2400 × g for 5 min. 50 μL of supernatants were incubated with 50 µL of 2X substrate working solution containing 5 mM Z-DEVD-AMC substrate and incubated for 60min at room temperature. Fluorescence was measured at 485/530 nm excitation/emission wavelength. Fluorescence signals were normalized for µg of total protein and the data were expressed as µM of cleaved substrate considering a cleaved substrate standard curve.

#### 2.9.4 Microscopic dysmorphic nucleus detection

To analyse variation in nuclear morphology, HepG2 cells were treated with PLGA-Cap or Cap for 24 h and then stained with Hoechst (1 mg/mL in PBS) (Merck, Milan, Italy) for 10 min. Stained nuclei were observed with Olympus IX83 fluorescent microscope and variation in nuclear morphology was analysed using ImageJ software.

#### 2.9.5 Western blot analysis

For western blot analysis, HepG2 cells were plated at density of 3.5 × 10^4^/cm^2^ and, after 24 h, treated with PLGA-Cap or Cap for indicated times and then mechanically harvested with RIPA buffer (20 mM Tris HCl, pH 7.5, 150 mM NaCl, 1 mM EDTA, 1 mM dithiothreitol, 0.1% sodium dodecyl sulphate, 1% triton X-100, 1 mM orthovanadate, and a cocktail of inhibitors (Merck). 50 µg of total proteins extract were separated on a 12% sodium dodecyl sulphate–polyacrylamide gel electrophoresis, then transferred to a PVDF membrane (Merk Millipore ltd, Italy) and incubated first with following mouse primary antibodies: anti-Bcl2 anti-Bax, anti-Caspase 3 anti-sequestosome-1 (p62), anti-microtubule-associated proteins 1A/1B light chain 3B (LC3) (Thermo- Fisher Scientific, Inc., Italy), anti-GAPDH (Santa Cruz Biothecnology), overnight at 4°C (dilution 1:1,000 in 1% non-fat dry milk in TBS-Tween 20 0.1%) and then with horseradish-peroxidase conjugated anti-mouse secondary antibody (Bio-Rad laboratories S.r.l, Milan, Italy) for 1 h in TBS-Tween 20 0.1%; finally, immunocomplexes were revealed using a chemiluminescence detection kit (Merck) according to the manufacturer’s instructions. The western blot images were obtained by using the iBright image system and analyzed using the iBright image software (Thermo Fisher Scientific, Inc.).

#### 2.9.6 Reactive oxygen species (ROS) detection

To detect ROS accumulation in HepG2 cells we performed a 2′,7′-Dichlorodihydrofluorescein diacetate (DCFH-DA) assay. HepG2 cells were plated at density of 8 × 10^4^/cm^2^ 24 h before stimulation and were treated at indicated concentrations of PLGA-Cap and free Cap for 6 h. After treatmets, cells were washed twice with PBS and incubated with DCFH-DA for 30 min at concentration of 10 µM in growing medium. Fluorescent signal were observed with Olympus IX83 fluorescent microscope and quantitative signal measured at an excitation wavelength of 485 nm and an emission wavelength of 530 nm. Fluorescence signals were normalized for µg of total protein and data expressed as fluorescence unit/protein concentration indicating normalized ROS level.

### 2.10 Cellular uptake by Confocal Laser Scanning Microscopy

The uptake of PLGA nanoparticles loaded with the Coumarin 6dye (C6 Loaded PLGA) by HepG2 cells was assessed by Confocal Laser Scanning Microscopy (CLSM) (STELLARIS 8 - Leica Microsystems, Wetzlar, Germany). HepG2 cells were seeded on plate at density of 8 × 10^4^/cm^2^ 24 h before stimulation. At each time point (1 h and 3 h), slides were washed with PBS and fixed with 4% Paraformaldehyde (PFA). The CLSM is equippend with a white light laser tunable between 440 and 790 nm for the excitation, while, a HC PL APO CS2 40x/1.10 water immersion objective was used for image acquisition. Emission signals were acquired with Power HyD detectors. The system was controlled using Leica Application Suite (LAS) X v4.3 software (Leica Microsystems, Wetzlar, Germany). The excitation wavelengths and detection windows for Coumarin 6-loaded nanoparticles were as follows: Hoechst 3,342 (excitation at 405 nm; detection range 420–455 nm) and Coumarin 6 (excitation at 464 nm; detection range 480–550 nm).

### 2.11 Statistical analysis

All the data shown in this study were presented as the mean ± SD or STDERR of three distinct investigations (unless otherwise indicated). The standard deviation (SD) of the mean is shown by the error bars. Data were statistically analyzed using Origin 2018 (OriginLab, United States) and GraphPad Prism 5.0 (GraphPad Software, Inc., CA, United States).

## 3 Results and discussion

### 3.1 Particles synthesis and characterization

PLGA nanoparticles were synthesised by single emulsion solvent evaporation method. The different parameters such as polymers concentration, the nature of the Cap and homogenization settings have been investigated in detail to obtain nanoparticles with the best characteristics in terms of dimension, yield, PDI and Cap loading.

In this work, 18 different conditions of Ultrasonication were tested, changing the parameters of amplitude, sonication duration (Time ON) and numbers of repetitions. Since amplitude and time are two important parameters that need to be carefully considered to obtain nanoparticles with optimal properties, we tested different conditions to select the appropriate settings. (Yan et al., 2022). In particular, the amplitude provides the necessary force to break the interface between the two phases formed during the synthesis (Water and Oil), which are immiscible with each other, and allow the formation of particles.

The duration of sonication is also significant as it determines the amount of acoustic energy delivered to the suspension. In addition, setting a non-continuous pulse, or a delay between repeated activations (time ON and OFF) prevents the polymer from overheating. (Ruiz et al., 2022). All tested settings are listed in Table 1.

**TABLE 1:** Optimization of Ultrasonication synthesis parameters.

Sample	Amplitude	Time ON (minutes)	Repetition	Time OFF (seconds)	Total Time (minutes)
1)	30%	2	5	30	10
2)	30%	1	10	30	10
3)	30%	5	2	30	10
4)	35%	2	5	30	10
5)	35%	1	10	30	10
6)	35%	5	2	30	10
7)	38%	2	5	30	10
8)	38%	1	10	30	10
9)	38%	5	2	30	10
10)	40%	2	5	30	10
11)	40%	1	10	30	10
12)	40%	5	2	30	10
13)	45%	2	5	30	10
14)	45%	1	10	30	10
15)	45%	5	2	30	10
16)	50%	2	5	30	10
17)	50%	1	10	30	10
18)	50%	5	2	30	10

**FIGURE 2:**
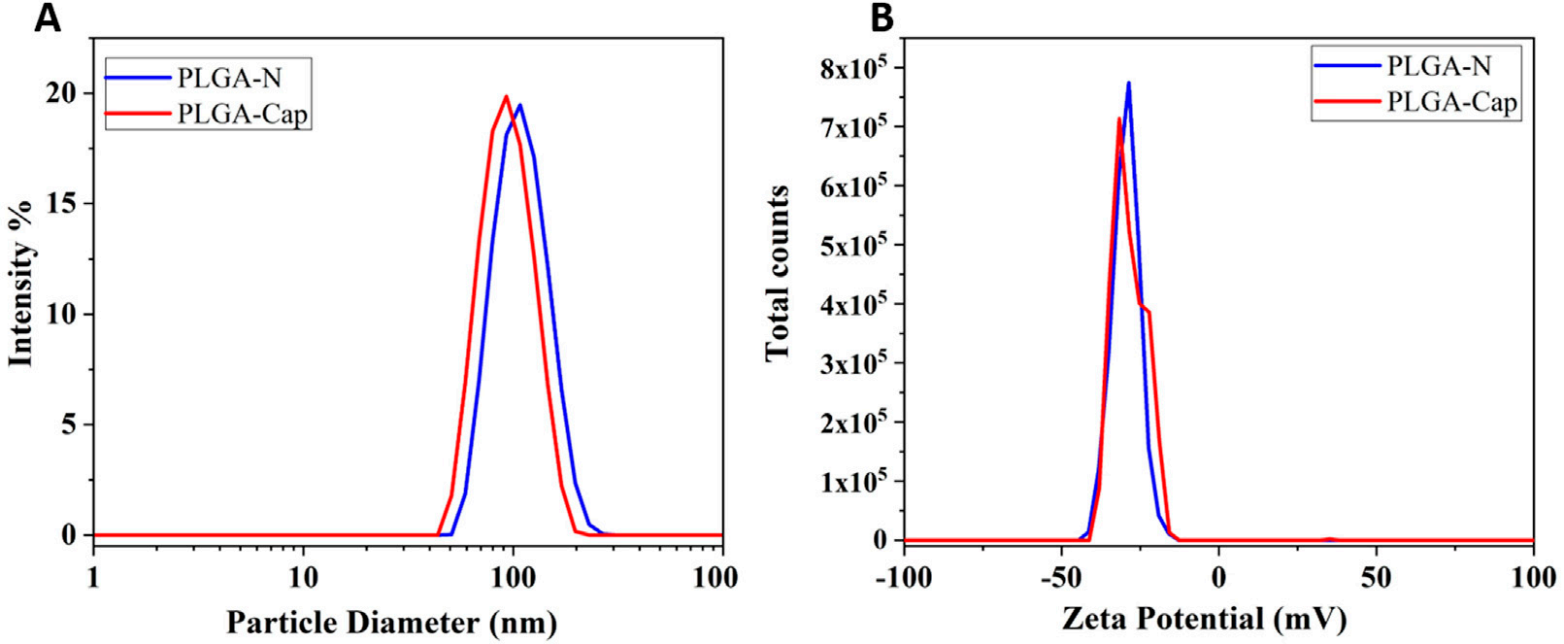
Characterization of the optimized particle synthesis by DLS. In blue and red lines were reported the hydrodynamic diameter **(A)** and the Zeta potential **(B)** measurement of PLGA-Naked and of PLGA- Cap respectively.

The optimization of the ultrasonic phase influences the properties of the particles in terms of size and PDI. Once all formulations were characterized by DLS, we report only the data of the particles that have a PDI <0.2 and a size that is in the ideal range of the order of 100 nm (Table 2). For this reason, the sample 1, 3 and 7 were excluded for PDI > 0.2 and sample 2 for size > 300 nm. Nanoparticle yield (Table 2) was assessed post-freeze-drying with 1.25% w/v trehalose. Small particle size hindered pellet formation during washing, causing losses in the supernatant and resulting in low yields. Consequently, samples 8, 9, and 15 were omitted from further analysis due to insufficient yield. The optimized synthesis process resulted in high-quality nanoparticles, characterized by diameters less than 120 nm and a highly uniform size distribution (PDI <0.2), as confirmed by DLS (Figure 2).

**TABLE 2:** DLS characterization of PLGA particles. The sample 1, 3-7 were excluded for PDI> 0.2 and sample 2 for the Size.

Sample	Z-average (Dh,nm)	PDI	Peak (Dh, nm)	Yield (%)
8)	143 ± 1.19	0.133 ± 0.355	156 ± 6.71	1.70
9)	151 ± 2.19	0.171 ± 0.021	181 ± 50.02	0.24
10)	107 ± 1.69	0.060 ± 0.017	115 ± 3.82	9.00
11)	111 ± 0.345	0.036 ± 0.046	117 ± 3.22	15.2
12)	107 ± 1.22	0.029 ± 0.025	112 ± 2.30	10.2
13)	114 ± 3.42	0.188 ± 0.060	114 ± 3.21	9.50
14)	119 ± 0.98	0.154 ± 0.039	130 ± 3.78	10.8
15)	114 ± 5.21	0.121 ± 0.118	116 ± 6.47	5.50
16)	130 ± 2.89	0.196 ± 0.042	134 ± 3.98	15.5
17)	117 ± 2.26	0.141 ± 0.056	123 ± 7.03	15.0
18)	123 ± 5.24	0.192 ± 0.077	124 ± 5.49	11.2

In particular, the PLGA-N nanoparticles have a diameter of 117 ± 3.16 nm and Zeta potential of −30.4 ± 0.81 while the PLGA-Cap have a diameter of 96.84 ± 3.95 nm and zeta potential of −26.8 ± 1.7 mV (Figure 2). The variation in the size of the loaded particles compared to the Naked ones is given by the hydrophobic interaction between the hydrophobic Cap molecule inside the core and the hydrophobic chain of polymer (Jäger et al., 2018). Our preparations stand out as they are significantly smaller than all other Cap loaded PLGA NPs reported in the literature. While previous studies have documented Z-average sizes ranging from 145–250 nm (Parashar et al., 2019; Fan et al., 2022; Malewicz et al., 2022) to as large as 3.9 µm (Kim et al., 2011) our samples achieve a markedly smaller size. This reduced dimension is a key advantage, as it directly influences crucial secondary properties such as targeting efficiency, degradation rate, and cellular uptake mechanisms (Sur et al., 2019). The stability of the PLGA-Cap nanoparticles was tested 7 months after production (see Supplementary Figure S1; Supplementary Table S1), indicating that no physicochemical modifications occurred during this long storage period.

**FIGURE 3:**
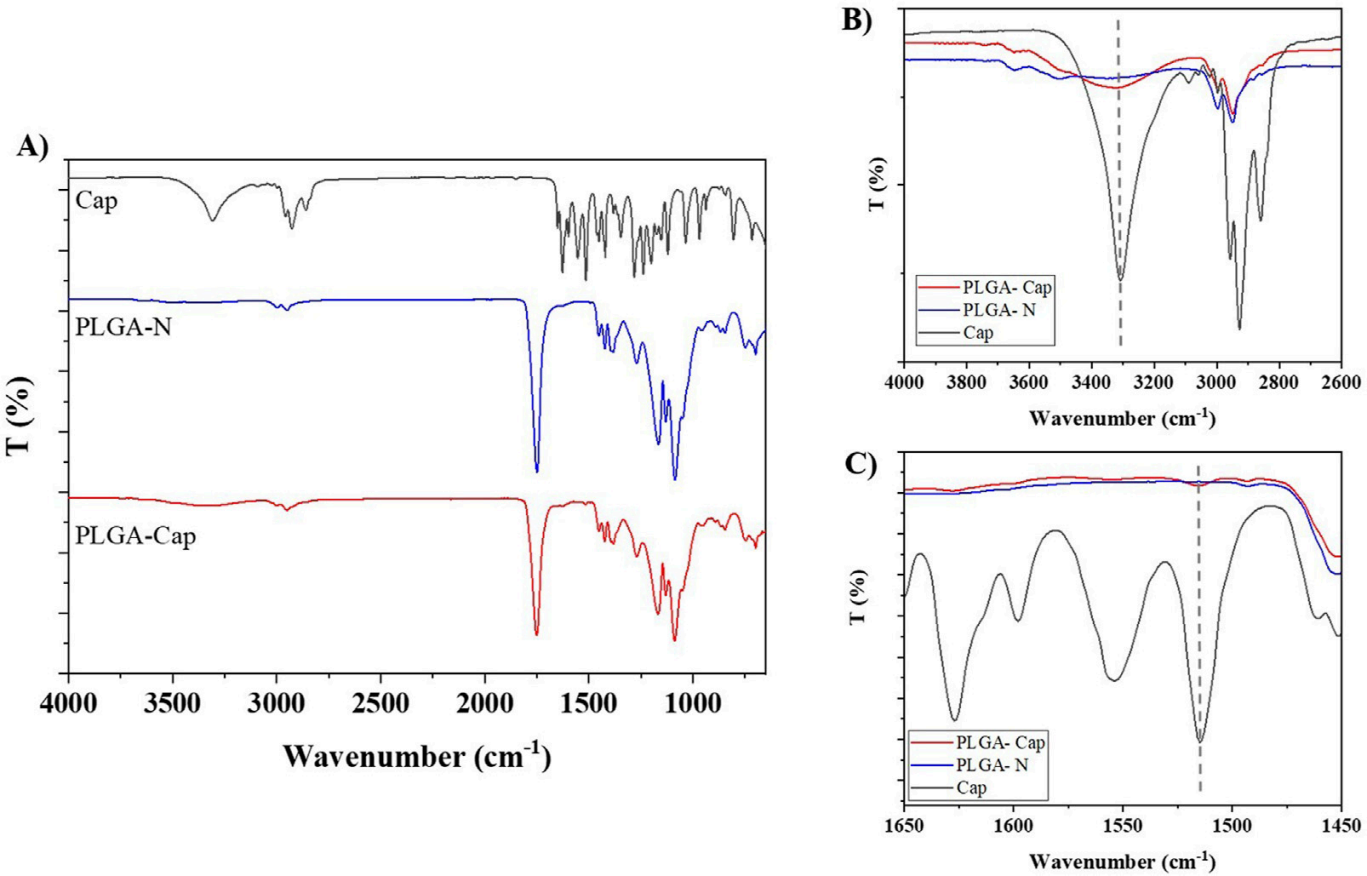
**(A)** Characterization of PLGA nanoparticles performed by FTIR-ATR. **(B,C)** insets of the characteristic Cap bands at 3,324 and 1,516 cm^−1^, respectively.

Fourier transform infrared spectroscopy (FTIR) was performed on free capsaicin, PLGA-N NPs and PLGA-Cap NPs to further confirm the encapsulation capability of the proposed carrier. As shown in the Figure 3, for the Naked NPs is possible to observe: a sharp peak at 1750 cm^-1^ due to the C=O stretching vibration of the ester group of the polymer; a very pronounced peak at 1,088 cm^-1^ which corresponds to C-O-C; and a band between 2,850 and 3,000 given by the stretching of C-H. (Zhou et al., 2013). Compared to naked ones, PLGA-Cap NPs have an extra band at 3,324 cm^−1^ (shifted compared to capsaicin alone which has it at 3,447 cm^−1^) which refers to an amino group (N-H) and a hydroxyl group. Another amino group is observed at 1,516 cm^−1^ that is characteristic of Capsaicin. (Mondal et al., 2019). A small peak at 1,629 cm^−1^ was attributed to olefinic C=O stretch, C=C stretch, and amide II (Giri et al., 2017). All the FTIR results are reported in the Table 3. The presence of analogous bands in the spectra of both free Cap and our PLGA-Cap confirms the successful encapsulation achieved with our formulation.

**TABLE 3:** Schematic representation of the characteristic peak of the Cap and PLGA obtained by FTIR analysis.

Molecule	Wavelength number (cm^-1^)	Group
Capsaicin	3,324	Amino group (N-H) band
	1,516	Amino group (N-H) band
	1,629	Olefinic C=O stretch, C=C stretch, and amide II
PLGA	1,088	C-O-C stretch
	1750	C=O stretching vibration of the ester group
	Band between 2,850 and 3,000	stretching of C-H

**FIGURE 4:**
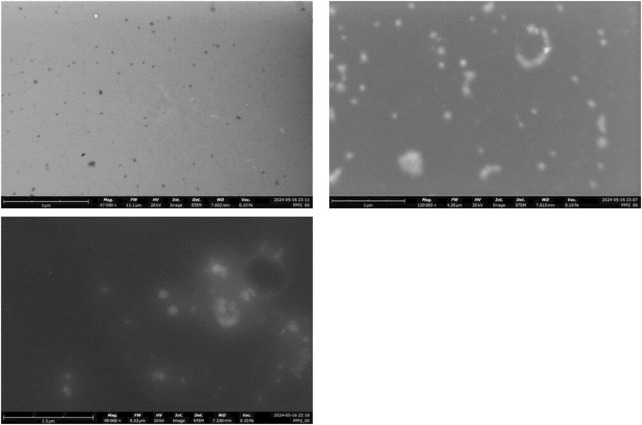
STEM characterization of the PLGA nanoparticles.

In order to obtain information about the morphology of the Nanoparticles, STEM analysis was conducted. The results suggest that the PLGA-Cap had a spherical shape as shown in the Figure 4. Encapsulation Efficiency (EE) and the Drug Loading (DL) were quantified by RP-HPLC as describe in the Methods section. The developed method allows the analysis of up to 1 µg of Cap. The different preparations obtained were characterized to obtain the value of EE% and DL%. As shown in the table below (Table 4) the best results were obtained following the last three protocols (samples 16, 17 and 18- the one that were synthetized with 50% of Amplitude). By combining the main characteristics of the formulation such as size, PDI and EE%, we select the synthesis protocol used for sample 17 for all further particle preparations (EE% of 74.41 ± 0.12 and a DL% of 12.40 ± 0.02).

**TABLE 4:** Characterization of the Cap-PLGA nanoparticles by RP-HPLC to obtain information of EE% and DL%. The best result is for the sample 17 that has the higher EE% and DL%.

Sample	EE%	DL%
10	12.46 ± 0.05	2.07 ± 0.01
11	30.93 ± 0.36	5.15 ± 0.06
12	20.99 ± 0.10	3.49 ± 0.02
13	37.98 ± 0.23	6.33 ± 0.04
14	36.96 ± 0.11	6.16 ± 0.02
16	64.07 ± 0.74	10.67 ± 0.12
17	74.41 ± 0.12	12.40 ± 0.02
18	74.14 ± 0.36	12.35 ± 0.06

**FIGURE 5:**
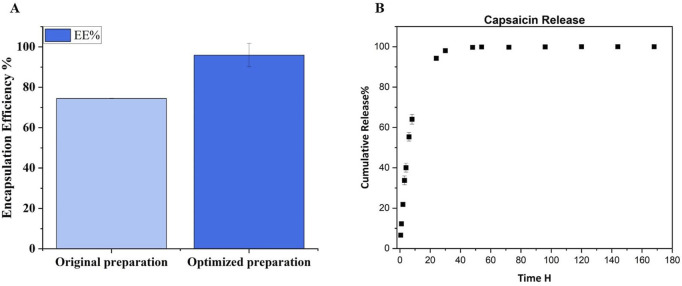
**(A)** EE% evaluation of original preparation and optimized preparation after the adjustment of centrifuge parameters. Was obtain an improvement of 21% respect the first preparation. **(B)** Cumulative release curve of Capsaicin from PLGA-NPs. The experiment was performed in Tween 20 buffer at 37°C with continuous agitation.

Once the sonication parameters have been chosen, we have optimized incubation, centrifuge conditions and washing times. Regarding the centrifuge condition, the initial centrifuge parameters were 10 min, 16,000 rcf at Room Temperature (RT); the optimization involves increasing the centrifugation speed and time with decreasing the temperature, 20,000 rcf and 4°C for 1 hour (as reported in the method section) in order to break down the particles better. So, we have achieved improved performance and encapsulation efficiency, obtaining an EE% of 95.91 ± 5.77 e DL% 16.23 ± 0.56. This result suggest that we have an improvement of 21% respect our first preparation, as it is shown by Figure 5A.

To the best of our knowledge, our preparation has the higher EE% for Cap loaded in PLGA NPs, compared to what is currently reported in the literature. Actually the Cap-loaded PLGA preparation with the highest EE% is 75% (Fan et al., 2022), so our preparation has an improvement of 21%. Other paper reported PLGA NPs loaded with Cap but the EE% is lower respect our results. As it is shown by the Table 5, in the other papers with Cap loaded in PLGA NPs the EE% does not exceed 50% (Kim et al., 2011; Parashar et al., 2019; Malewicz et al., 2022). Lipid carriers demonstrated a comparable encapsulation efficiency (EE%) to our findings, likely due to their inherent ability to facilitate the internalization of capsaicin, a hydrophilic molecule. However, their particle size was larger than that observed in our samples (Qi et al., 2021; Arunprasert et al., 2022). The importance to have small nanoparticles depends on the passive targeting mediated by the enhanced permeability and retention (EPR) mechanism witch allow the NPs penetration in the cancer cells (Zhou et al., 2017; Dutt et al., 2023).

**TABLE 5:** Summary of the current information in literature about size, EE% and DL% for Cap delivery by different carriers.

Carriers type	Carriers materials	Z-average nm	EE%	DL%	Case study	Ref
PLGA-Cap	Cap-loaded PLGA NPs	96.84±3.95	95.91 ± 5.77	16.23 ± 0.56	Hepatocellular carcinoma	Our proposal
HM PLGA@Cap NPs	Hybrid membrane (HM) camouflaged PLGA nanosystem for Cap delivery	120	75	-	Breast cancer	Fan et al. (2022)
Cap-PLGA	PLGA NPs for Cap delivery	201 ± 6.1	50±2.1	24.72±1.2	Lung Cancer	Parashar et al. (2019)
Capsaicin-NPs	Capsaicin-loaded PLGA NPs	247 ± 1.11	16.57	5.8	Acute itch and Heat pain	Malewicz et al. (2022)
Cap-PLGA nanoprecipitation method	Oil/water 1:2 Oil/water 1:5	162 ± 3 150 ± 3	25 20	- -	- -	Kim et al. (2011)
Cap-PLGA single emulsion method	Oil/water 1:10 Oil/water 1:2	346 ± 20 3.9 ± 1 µm	12 49	- -	- -	Kim et al. (2011)
CAP/GA-sHA-DOX NPs	CAP and DOX in glycyrrhetinic acid (GA) modified sulfated-HA (sHA) nanocarriers	253 ± 3.25	72.08±0.44	12.02±0.08	Hepatocellular carcinoma	Li et al. (2021b)
CAP/DOX@NPs	CAP and DOX loaded mPEG-αPLA	98	21.2	4.3	Hepatocellular carcinoma	Yan et al. (2022)
CAP-in-βCD	Capsaicin-in-β-cyclodextrin (βCD) inclusion complexes	181 ± 36.23	38.65 ± 3.7	1.65 ± 0.16	Breast cancer	Abdelnabi et al. (2021)
Caps-Cur/GA&Gal-Lip	Modify liposomes for co-delivery of curcumin and capsaicin, with glycyrrhetinic acid and galactose as ligands	138.97 ± 2.97	89.95 ± 0.05	2.04 ± 0.05	Hepatic cancer	Qi et al. (2021)
Cap-loaded NLC	Nanostructured lipid carriers (NLCs) loaded with Cap	180.3 ± 0.6	99.47 ± 0.04	15.18 ± 5.12	For transdermal delivery of Cap	Arunprasert et al. (2022)
Cap/MPEG-PCL-NPs	CAP-loaded methoxy poly (ethylene glycol)-poly(ε-caprolactone) nanoparticles	82.54 ± 0.5	81.5 ± 0.9	14 ± 0.13	-	Peng et al. (2015)
CAP:BSA NPs	CAP-loaded albumin nanoparticles	205 ± 25nm	98.3 ± 7.4%	-	-	De Freitas et al. (2018)
NC loaded	Capsaicin-loaded chitosan Nanocapsule	261 ± 32	92±2	6.5 ± 0.14	Tight junctions of Epithelial cell	Kaiser et al. (2015)

With the same RP-HPLC method used for EE% estimation, the Cap released from our NPs was evaluated, as described in the methods section, by incubating the PLGA-Cap NPs in the release buffer solution (PBS at pH 7.4 with 0.3% w/v of Tween 20) at 37°C for a week. As seen in Figure 5B, the Cap has a fast release from PLGA-NPs, consistent with prior research on similar preparations. 64% is released by 8 h, 94.65% by 24 h, and near-total release (99.7%) by 48 h, indicating full release within 2 days. The kinetic of release of Cap from PLGA was observed to have the same rate also in other papers where in some cases it takes 24 h to reach 81% (Parashar et al., 2019), but at most 100% of the release is reached in 72 h (Kim et al., 2011; Malewicz et al., 2022).

**FIGURE 6:**
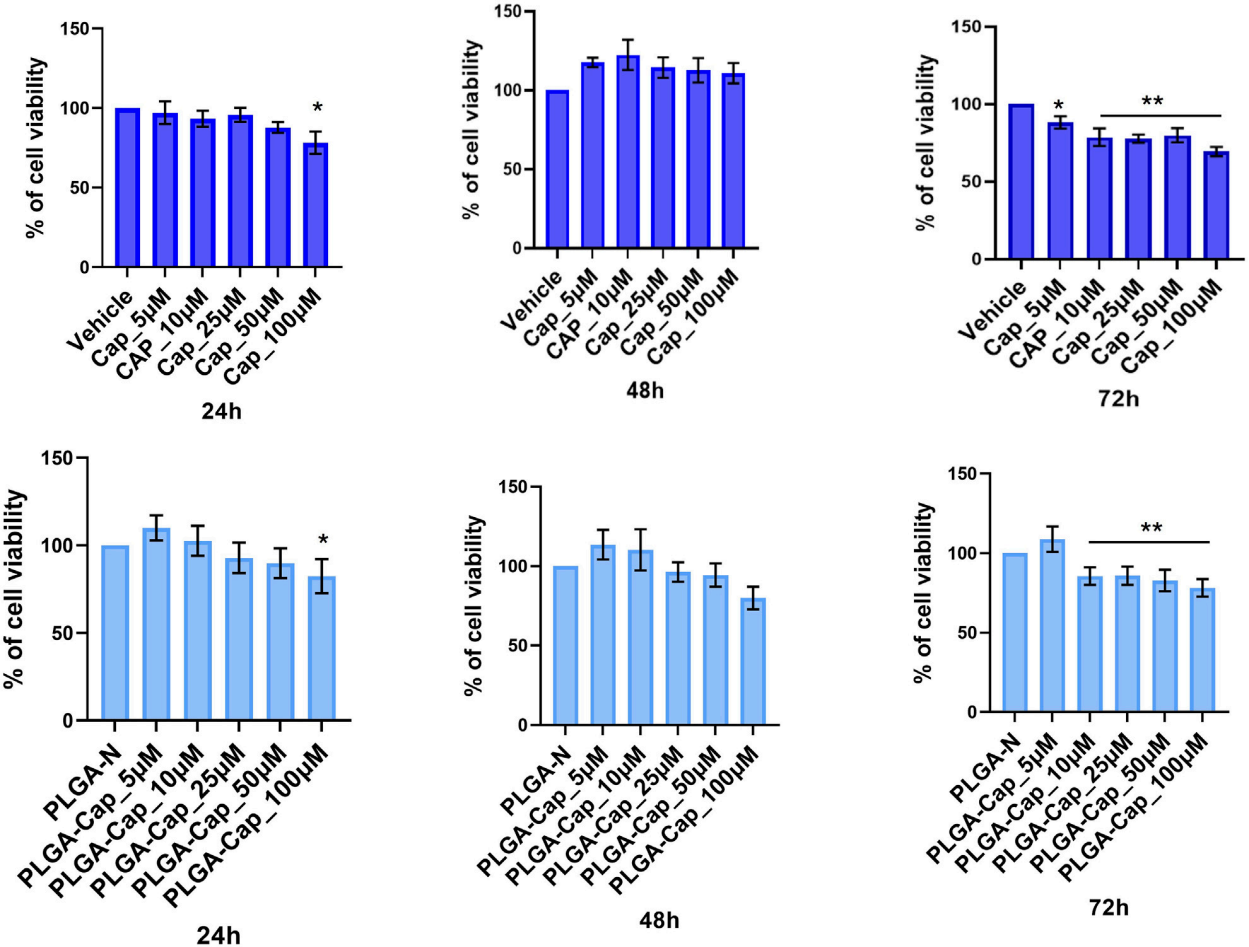
MTT viability assay. HepG2 cell line was treated for 24 h, 48 h, and 72 h with different concentrations of PLGA-Cap or free Cap and then the MTT assay was performed. Data were expressed as fold change (mean ± STDERR) *versus* PLGA-N or Vehicle (DMSO); data were calculated from three independent experiments, each in triplicate. Both DMSO and PLGA did not induced cell viability reduction at any concentration used. *p value <0.05, **p value <0.01 *versus* PLGA-N for PLGA-Cap and the vehicle for free Cap.

We also evaluated the *in vitro* biological effect of our preparations on HepG2 cell line. First, we performed cell viability MTT assays in a range of concentrations of 100–300 μM, taking in account that reported calculated IC_50_ for Cap at 24 h was very heterogeneous, ranging from 365 μM (Impheng et al., 2014) to 150 μM (Bort et al., 2017), depending on experimental conditions employed. In accordance with previous works, after seeding cells at density 2.9 × 10^4^/cm^2^, we observed an IC_50_ at 24 h of 265 ± 8 μM for free Cap. We also measured IC_50_ at 24 h for PLGA-Cap and we found a value of 276 ± 17 μM. These findings indicated that free Cap and PLGA loaded Cap had similarly effects on cell viability. In the attempt to highlight eventual differential cell responses in a sub-toxic range of concentrations, we next performed MTT assays at a low cell density (1.6 × 10^4^/cm^2^) with Cap concentrations between 5 and 100 μg/mL for 24 h, 48 h, and 72 h (Figure 6). At 24 h, a slight but significant reduction of cell viability was registered only at 100 µM. At 48 h, we observed a slight increase of cell viability, more evident for free Cap. This unexpected finding might be due to a pro-proliferative effect of Cap at low doses, as already reported in other *in vitro* systems, when a stimulation longer than 24 h was applied (Malagarie-Cazenave et al., 2009; Zhai et al., 2018; Khonglim et al., 2024). At 72 h, the viability-reducing effect predominated: we observed that PLGA-Cap reduced cell viability with a similar trend with respect to free Cap, with a significant reduction in the concentration range 10–100 µM for PLGA-Cap and 5–100 µM for free Cap. On the whole, these data confirmed a similar behaviour of PLGA-Cap with respect to free Cap in exerting an inhibitory effect on HepG2 cell viability.

**FIGURE 7:**
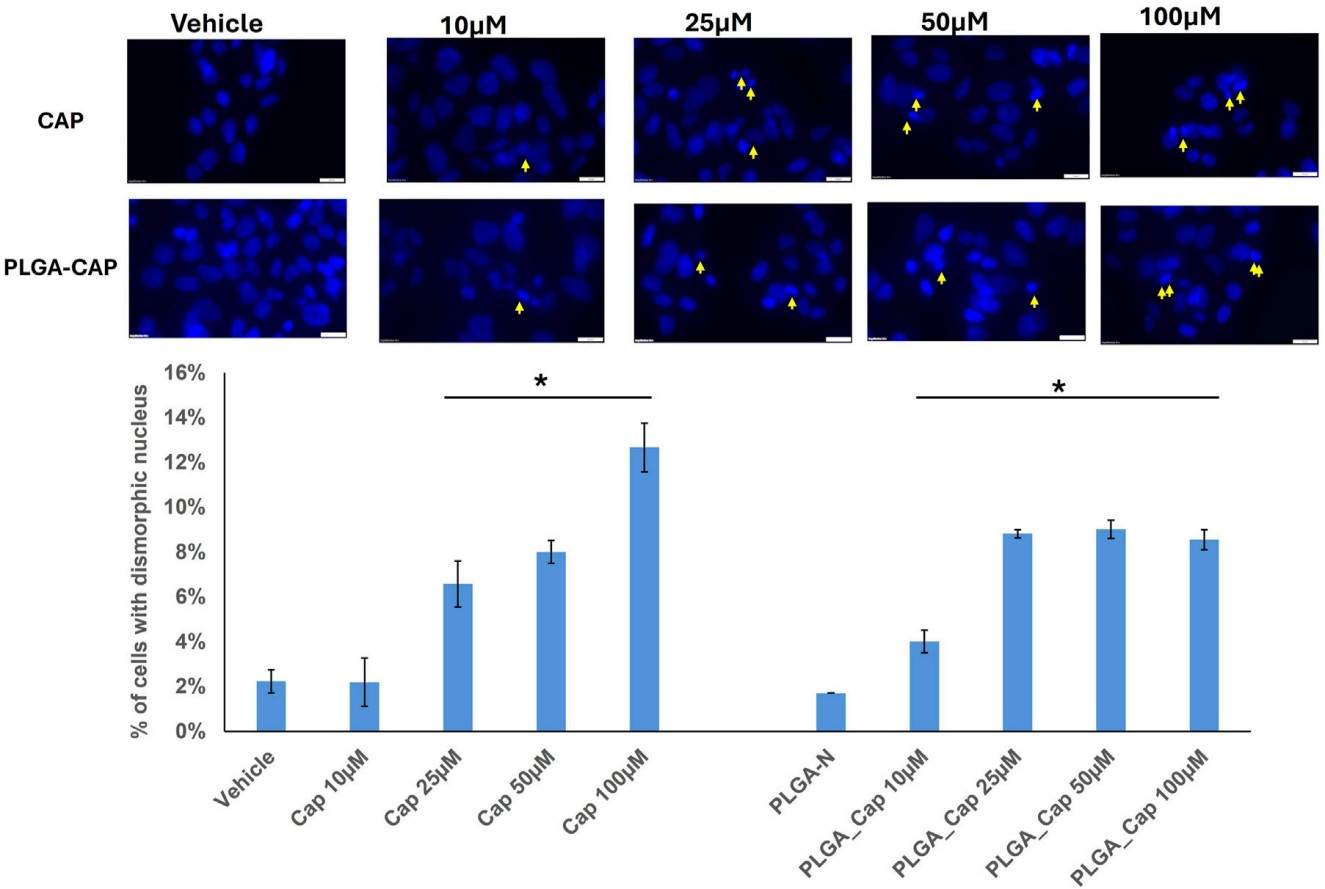
Dysmorphic nuclei assay. Dysmorphic nuclei of HepG2 cells stained with Hoechst were counted after 24 h of treatments with PLGA-Cap and free Cap. Representative images of stained nuclei (magnification ×40) for each treatment are shown. The mean number (±STDERR) of dysmorphic nuclei is reported as percent of total nuclei. *p value <0.5 *versus* Vehicle or PLGA-N.

To better characterized the biological effect of PLGA-Cap preparations we observed nuclei morphology after treatments with doses already employed in the MTT assays, i.e. 10–100 μM, for 24 h, with the aim to observe early nuclear events that could be indicative of cell death induction. Nuclei of HepG2 cells treated for 24 h with PLGA-Cap or free Cap were stained with Hoescht and then visualized by fluorescence microscopy. We observed a slight increase of dysmorphic nuclei for both treatments compared to the respective vehicles (Figure 7), however this increase was significant in the range 25–100 µM for free Cap and in the range 10–100 µM for PLGA-Cap.

**FIGURE 8:**
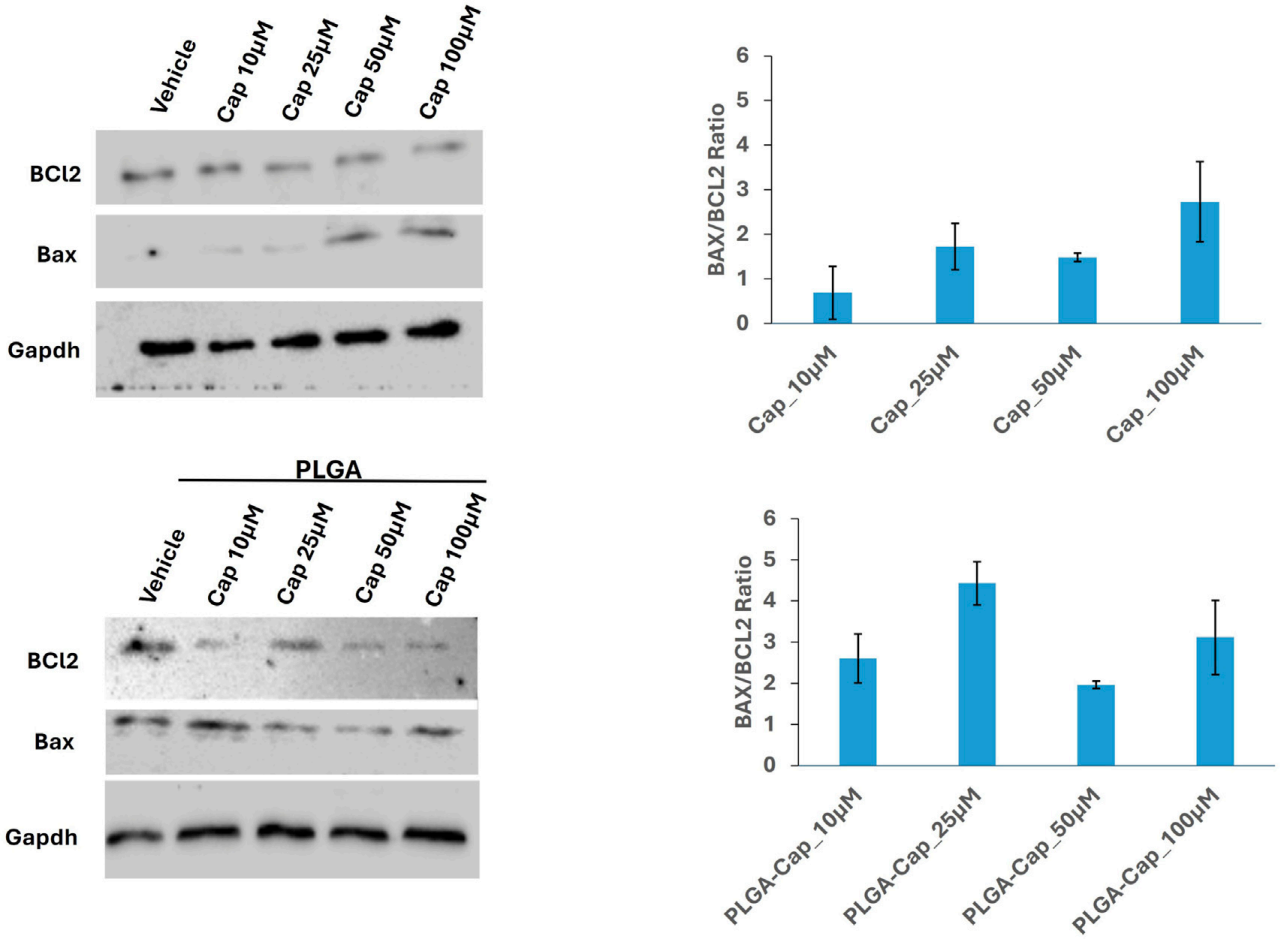
Apoptosis induction. Representative western blots showing the expression of apoptotic markers, Bcl2 and Bax in samples consisting of 50 μg of total protein extracts obtained from HepG2 cells treated for 24 h with PLGA-Cap or free Cap. The Bax/Bcl2 ratio, calculated considering two independent experiments, was reported in graph (mean ± STDERR).

Considering that alteration of nuclear morphology together with nuclear fragmentation is an event that occurs in different cells death processes, including apoptosis (Ziegler and Groscurth, 2004), we investigated apoptosis induction by evaluating the level of proteins having a crucial role in apoptosis (Singh et al., 2019), in the same conditions employed for nuclear visualization. Thus, we analysed Bcl2 and Bax proteins, two markers involved in intrinsic pathway of apoptosis. In particular, the Bax/Bcl-2 ratio determines the cell fate in life or death in response to an apoptotic stimulus; an increased Bax/Bcl-2 ratio is central to induce cell death and loss of cell resistance to apoptosis (Vaskivuo et al., 2002). Western blot analyses revealed that in HepG2 cells, PLGA-Cap or free Cap were both able to slightly increase Bax level and reduce Bcl2 level. As a consequence, the Bax/Bcl-2 ratio increased for both treatments (Figure 8), suggesting that not only free Cap, but also PLGA-Cap similarly was inducing apoptosis.

**FIGURE 9:**
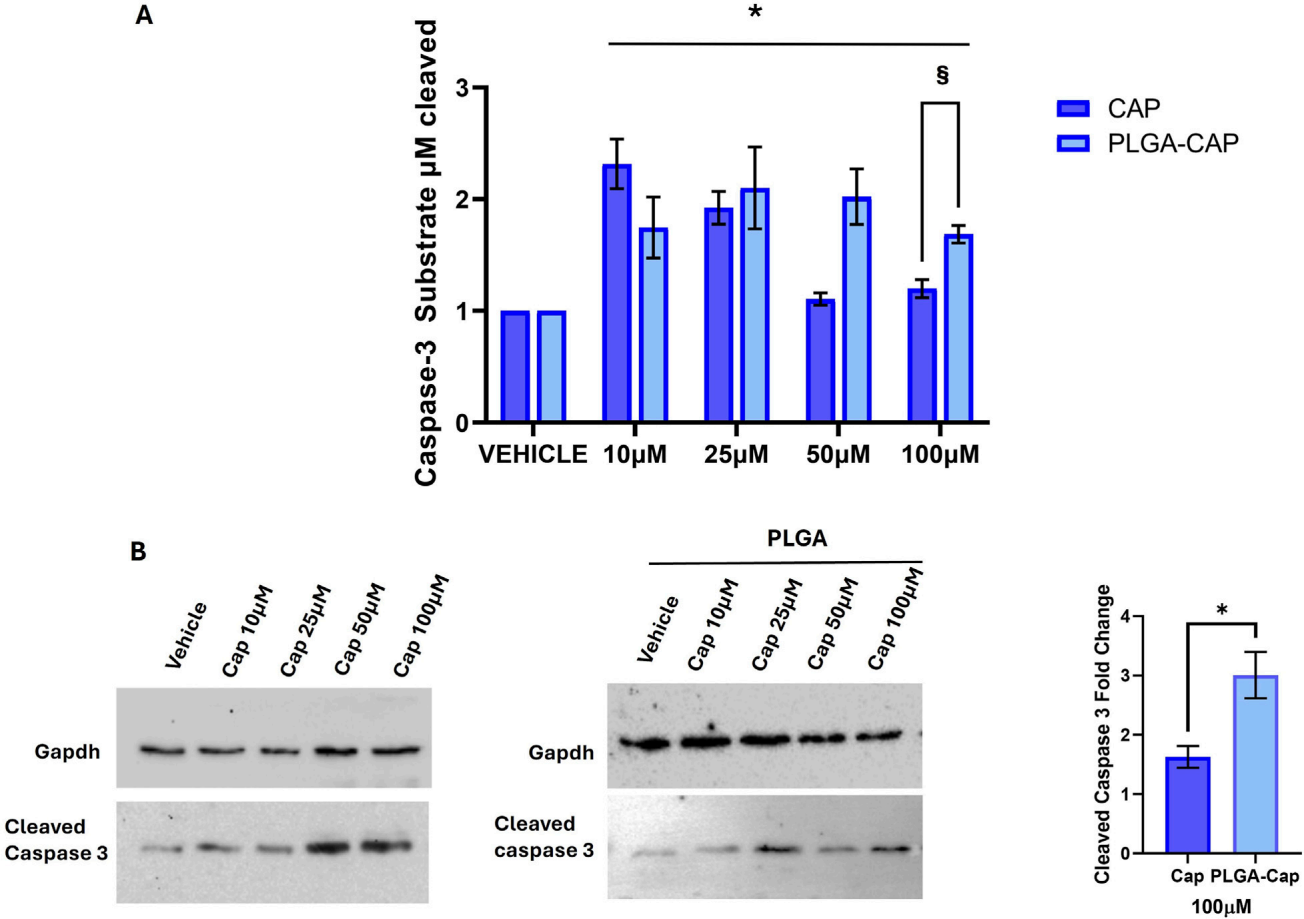
Caspase 3 activation. **(A)** Caspase 3 activity assay was performed after 6 h of treatments with Cap or PLGA-Cap; the activity is calculated as µM of caspase 3 substrate cleaved respect to standard curve, then data are expressed as fold change vs. vehicle (DMSO for Cap and PLGA-N for PLGA-Cap) considering three independent experiments. *p value <0.01 *versus* vehicle, § < 0.05 *versus* free Cap. **(B)** Representative Western blots of cleaved caspase 3 protein level after treatments of 48 h with PLGA-Cap or Cap. The fold change at 100 μM reported in the graph is referred to three independent experiments *p value <0.05 *versus* free Cap.

To obtain an accurate quantitative measurement of apoptosis induction, we also evaluated the enzymatic activity of caspase 3, an enzyme crucial in apoptosis programmed cell death (Porter and Jänicke, 1999). Given the high sensibility of the method to detect caspase 3 activity, we were able to observe a significant caspase 3 activation after 6 h of treatment with both free Cap and PLGA-Cap. We registered an effect that was higher at low concentration of free Cap, 10 µM and 25 μM, respect to concentrations of 50 μM and 100 µM (Figure 9A). In the presence of PLGA-Cap, caspase 3 activity increased at 10 µM of PLGA-Cap and maintained the same activation level at higher concentrations (25, 50, and 100 µM) (Figure 9A). Furthermore, at 100 µM we detected a significant higher increase of caspase 3 activity of 40% in PLGA-Cap respect to cells treated with free Cap. To support data from caspase 3 activity assay, we analysed, by western blot, how the cleaved and active form of caspase 3 accumulated into HepG2 cells. In this case, the treatment was prolonged tor 48 h to better visualize the caspase 3 protein level. We observed an increase of cleaved caspase 3 protein level both with PLGA-Cap and free Cap (Figure 9B); at 100 µM this increase was significantly higher of 46.7% in samples treated with PLGA-Cap than in samples treated with free Cap (Figure 9B), according with caspase 3 activity. Finally, we were interested also to evaluate another desirable property of the encapsulated Cap as potential anti-cancer drug, i.e., the ability to modulate autophagy. Indeed, several works reported that Cap was able to induce autophagy in several cancer cell lines, including HepG2 cells, at concentrations around 100 μM (Chen et al., 2016). Our data (Supplementary Figure S3) suggested that, at the experimental conditions at which we observed apoptosis, we also registered a moderate autophagic response in PLGA-Cap-treated cells.

**FIGURE 10:**
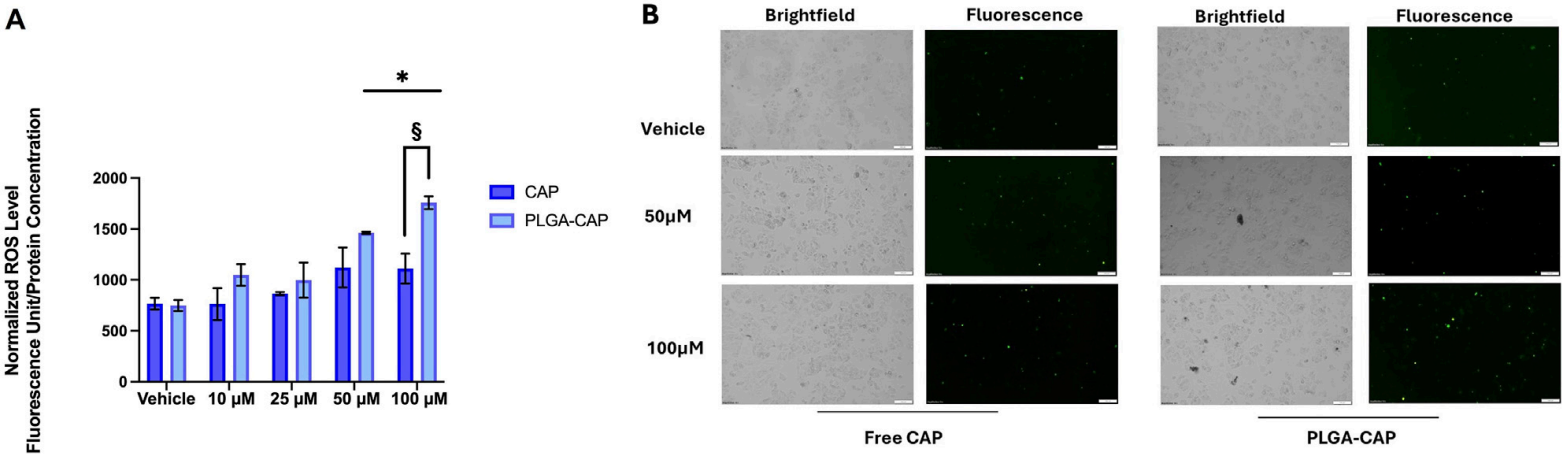
Oxidative stress assay. **(A)** Quantitative analysis of total DCFH-DA fluorescence levels detected after 6 h of treatments with Cap or PLGA-Cap in total cell lysates; data are expressed as mean ± STDERR of three independent experiments. *p value <0.05 and §p < 0.05 PLGA-Cap *versus* free Cap. **(B)** Rappresentative images of DCFH-DA fluorescent signals and brightfield fields (magnification ×10).

Considering that apoptotic induction *in vitro* by Cap is often mediated by ROS generation (Hacioglu, 2022; Zhang, R et al., 2008; Joung et al., 2007), we evaluated ROS level in presence of PLGA-Cap and free Cap. HepG2 were treated for 6 h with PLGA-Cap or free Cap and then ROS levels were analyzed by measure DCF fluorescence in Hepg2 cells stained with DCFDA. HepG2 increased DCFH-DA fluorescence accumulation as conseguence of ROS production in presence of both PLGA-Cap and free Cap (Figure 10); at the highest concentration tested (100 µM), ROS accumulation was significantly more induced in PLGA-Cap than in free Cap samples. In particular, we observed an increase of 58% at 100 µM of ROS levels in PLGA-Cap respect to free Cap samples. These findings indicated that an efficient ROS generation could be at the bases of apoptosis induction in HepG2-Cap-treated cells.

Very often, to improve the bioavailability, stability and pharmacokinetics of Cap, it is encapsulated in NPs of different nature and size. To assess the anticancer effects of these Cap formulations, their action on the apoptotic pathway involving Bax/Bcl2, caspase-3 expression and oxidative stress is frequently analysed (Merritt et al., 2022). By western blot it was found that Cap encapsulated in PLGA-PEG-NP increased caspase 3 levels in carcinoma lung cells isolated from rats (Parashar et al., 2019). Chitosan nanocapsules containing Cap also activated caspase 3 in T24 bladder cancer cells (von Palubitzki et al., 2020). Moreover, works by Elkholi et al. (E Elkholi, 2014) and Hazem et al. (Hazem et al., 2021) revealed that Cap loaded in trimethyl chitosan nanoparticles (TMC-NP) triggered apoptosis in HepG2 cells. Specifically, the researchers demonstrated that this formulation reduced the expression of the pro-survival gene Bcl-2 and increased that of the pro-apoptotic gene Bax. Furthermore, immunocytochemistry highlighted strong staining for caspase3 in cells treated with Cap-TMC-NP. Apoptosis was more evident when cells were treated with Cap-TMC-NP (100 µM) than with free Cap. (E Elkholi, 2014; Hazem et al., 2021). In HepG2 cells capsaicin loaded solid lipid nanoparticles (SLNs) induces apoptosis by ROS generation and loss mitochondrial membrane potential. (Kunjiappan et al., 2021). Overall, these results are in agreement with the biological effects induced by our preparation of PLGA-Cap, which increased the expression and activity of caspase 3, ROS generation and modulates the Bax/Bcl-2 ratio. Considering that PLGA nanoparticles have the ability to increase stability of loaded compounds and protect them from degradation (Rezvantalab et al., 2018; Yang et al., 2024), our results suggested that PLGA nano particles might have the ability to improve the effect of free Cap, possibly shielding Cap molecules from rapid cellular metabolization and inactivation processes.

**FIGURE 11:**
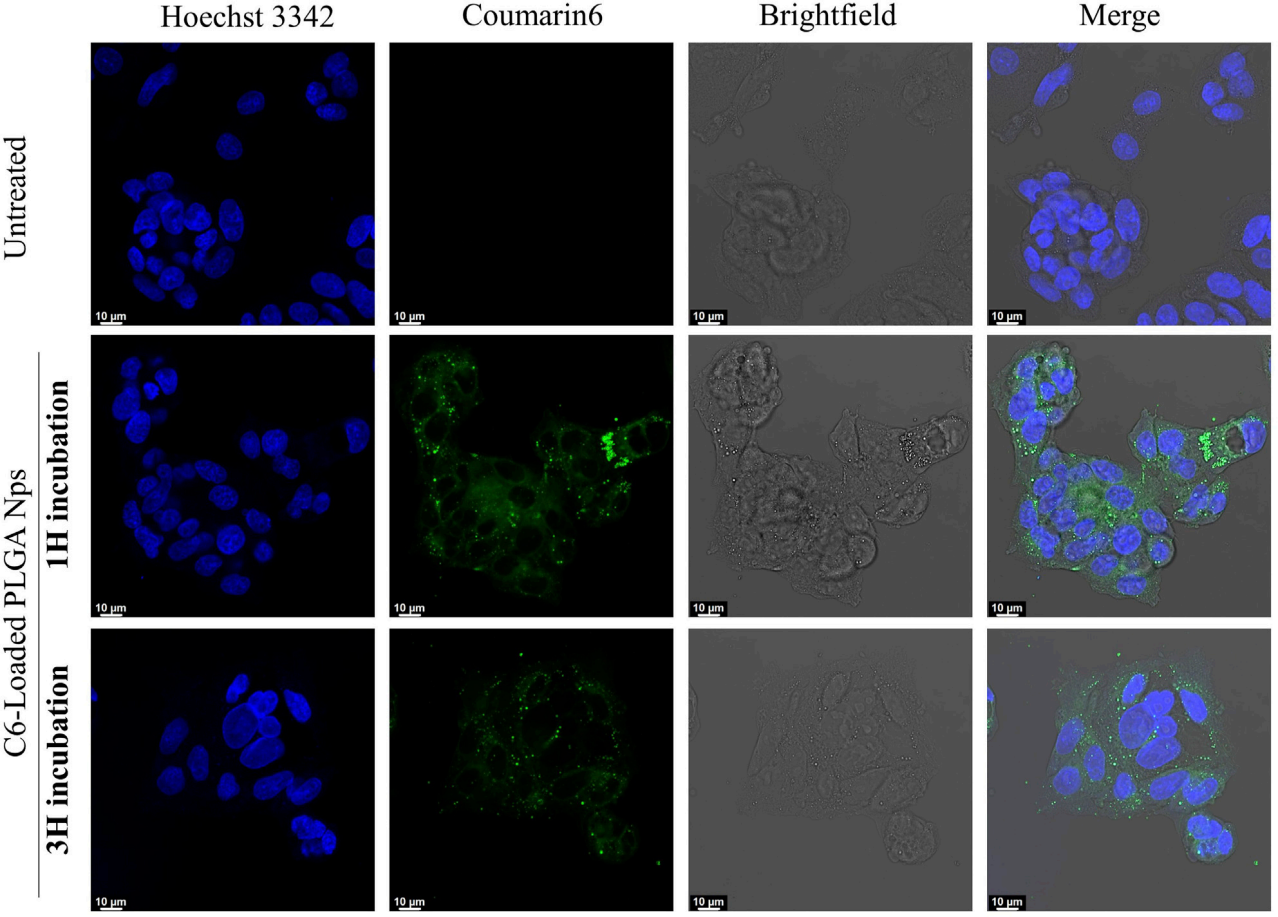
CLSM images of HepG2 cells uptake. Control (untreated) cells are compared with those incubated with C6-loaded PLGA nanoparticles for 1 and 3 h. The nuclei are stained blue, while the nanoparticles appear in green.

Finally, to further confirm the cellular uptake and intracellular transport capabilities of our PLGA nanoparticles, we investigated the intracellular distribution of C6-loaded fluorescent PLGA particles in HepG2 cells using CLSM (Vanni S et al., 2025). Fluorescence microscopy revealed rapid and substantial internalization of the PLGA nanoparticles, evidenced by a strong green fluorescent signal distributed throughout the cytoplasm within just 1 hour of incubation (Figure 11; Supplementary Figure S2).

## 4 Conclusion

This study aimed to develop PLGA nanoparticles for efficient capsaicin encapsulation. An optimization process, involving the synthesis of eighteen distinct samples, was performed to identify parameters yielding optimal nanoparticle characteristics, including minimal Z-average diameter, low polydispersity index, controlled surface charge, and maximized drug loading and encapsulation efficiency. The optimized formulation achieved a 96% EE%, a 21% improvement over the existing literature. *In vitro* analyses in HepG2 cells, comparing the pro-apoptotic effects of free capsaicin and capsaicin-loaded PLGA nanoparticles, indicated that both free Cap and PLGA-Cap induced apoptosis. Quantitative assay of caspase-3 activity showed that activation was detected even at 10 µM of free Cap and PLGA-Cap. However, at 100 μM, PLGA-Cap was slightly more effective (of 40%) in enhancing caspase 3 activation compared to free Cap. In the same way, PLGA-Cap induced more oxidative stress (of 58%) at 100 µM then free Cap. Our results validate the pro-apoptotic potential of Cap, suggesting that encapsulation within PLGA nanoparticles could boost its effectiveness. While further optimization is needed to maximize its anticancer effects, our current formulation also offers particular advantages for two key reasons: firstly, it paves the way for combination therapies with chemotherapeutic agents for enhanced anticancer action; and secondly, it could significantly alleviate the oral and gastric irritation, along with the burning sensation, well-known side effects of unformulated Cap administered orally or intravenously.

## Data Availability

The original contributions presented in the study are included in the article/Supplementary Material, further inquiries can be directed to the corresponding authors.
