# Supplementary material for: PLGA nanoparticles for capsaicin delivery: enhanced encapsulation efficiency and pro-apoptotic activity in HEPG2 cells
Source: Front Bioeng Biotechnol. 2025 Jul 18;13:1617022. doi: 10.3389/fbioe.2025.1617022 (PMC12313566; doi:10.3389/fbioe.2025.1617022)
Supplement: Supplementary file 1 [file DataSheet1.docx]

Supplementary Material

# Stability test of PLGA-Cap Nanoparticles

PLGA–CAP NPs stability was confirmed by DLS measurements. The nanoparticles were measured freshly prepared and then freeze-dried and stored at -20 °C for 7 months. The measures were repeated after 7 months to monitor the size variations concerning the initial NPs size, respectively. As shown in the table below a slight increase of size NPs, only 10 nm, occurred after this long period. Also in this case PDI remains <0.2.

**Supplementary Table 1**. Comparison of size and PDI between the PLGA-CAP NPs fresh prepared and after 7 months of storage to evaluate the NPs stability.

| **Sample** | **Z-average (Dh, nm)** | **PDI** | **Peak ( Dh, nm)** |
| --- | --- | --- | --- |
| Fresh prepared PLGA-CAP NPs | 101 ± 0.85 | 0.05 ± 0.01 | 107 ± 1.89 |
| 7 months age PLGA-CAP NPs | 111 ± 0.79 | 0.09 ± 0.02 | 120 ± 0.8 |


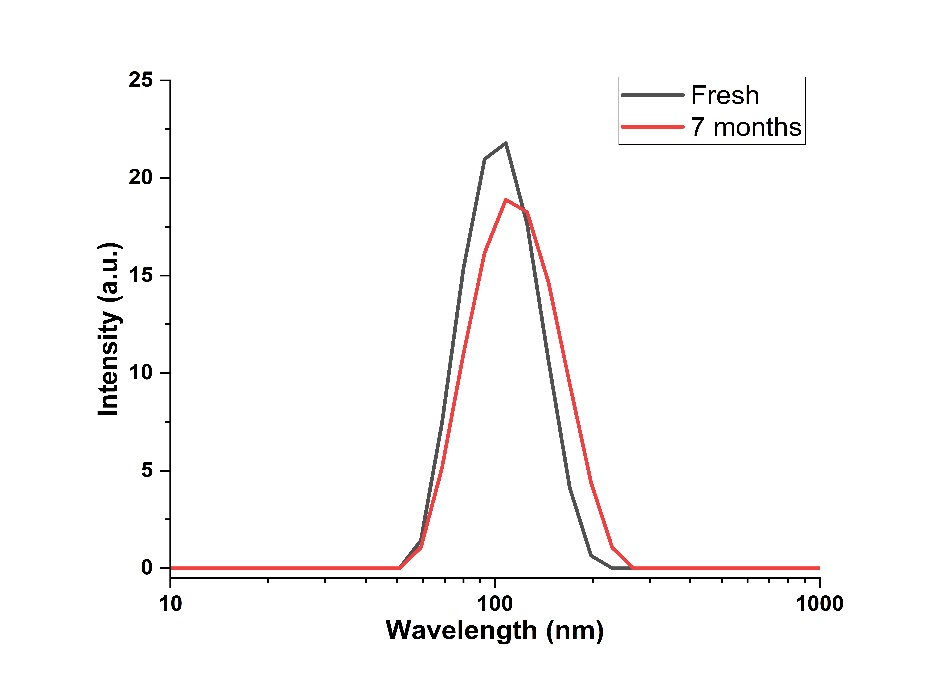


**Supplementary Figure 1.** The PLGA-CAP NPs size measurements by DLS fresh prepared (fresh, grey) and after seven months of storage (7 months, red).

Also the EE% was evaluated after 7 months with respect to the freshly prepared NPs formulations. For the quantification was used the same protocol reported in the methods section established for the evaluation of the EE% of the freshly prepared NPs. The EE% remain stable suggesting that no physical-chemical modifications occurred during this long period of storage (data do not shown).

# Cellular uptake of PLGA Nanoparticles

The 3D confocal fluorescence projections of HepG2 cells treated with C6-loaded PLGA nanoparticles clearly demonstrate the rapid and significant uptake of these carriers. Within just one hour of incubation, the fluorescent nanoparticles permeate the entire cytoplasm, confirming the impressive efficiency of PLGA vectors in delivering bioactive molecules such as capsaicin.


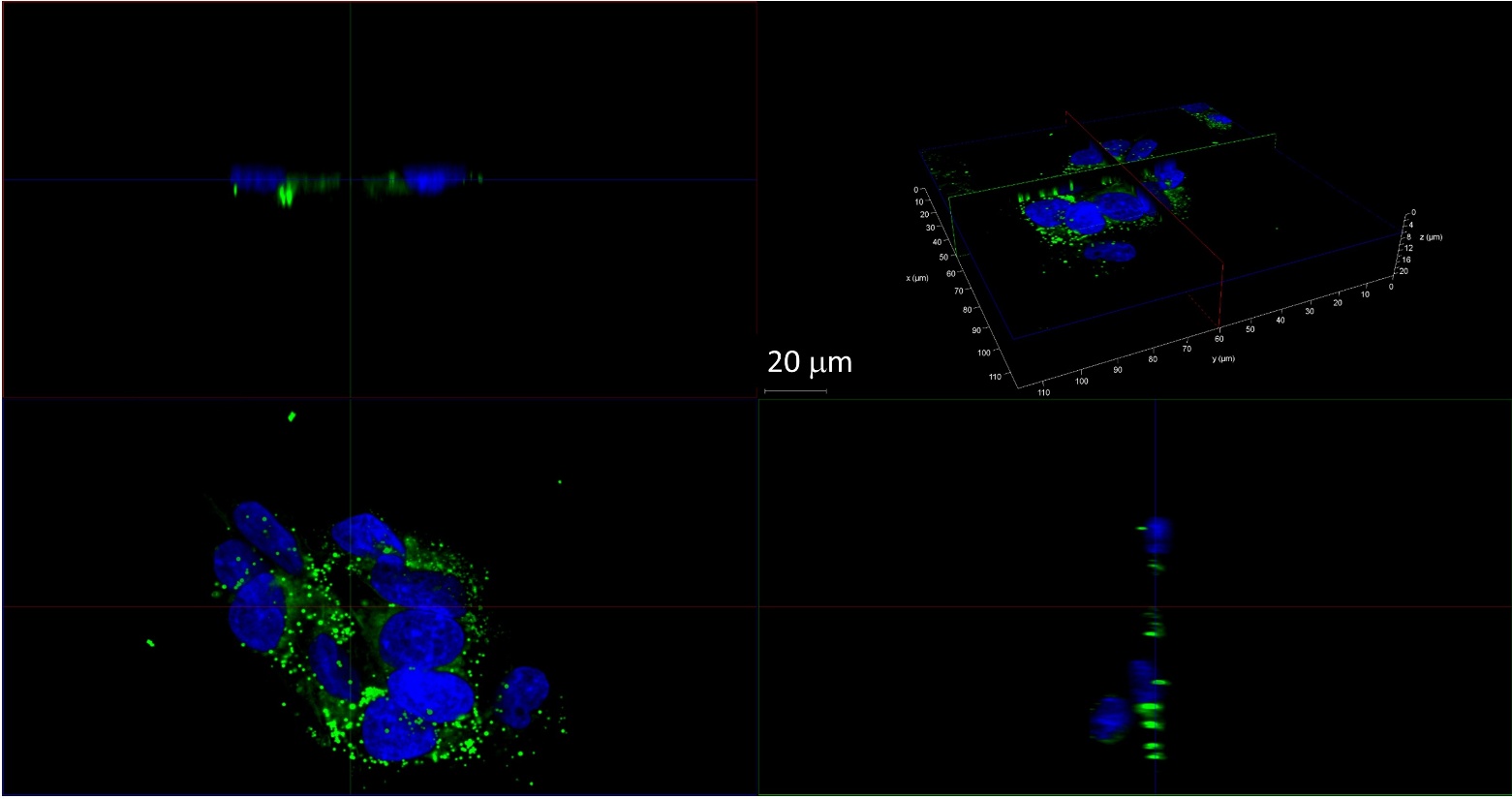


Supplementary Figure 2 Confocal z-stack reconstruction of HepG2 cells incubated with fluorescent PLGA nanoparticles. Nuclei are stained with DAPI (blue), while green green labelled nanoparticles are distributed throughout the cytoplasm.

# Autophagy modulation by PLGA-Cap

We verified the ability of PLGA-Cap to modulate autophagy by analysing two autophagic markers (LC3 and p62) in Western blot. We found that both free Cap and PLGA-Cap, at 100 μM, slight reduced p62, a protein involved in sorting of ubiquitinated proteins to the autophagosome, probably indicating a moderate increase of autophagy. To confirm this observation, we evaluated the level of LC3, another marker of autophagy. In cell treated with PLGA-Cap, at 100 μM, we observed a slight increase of the LC3 type II, which indicated an increment of the autophagosome formation.


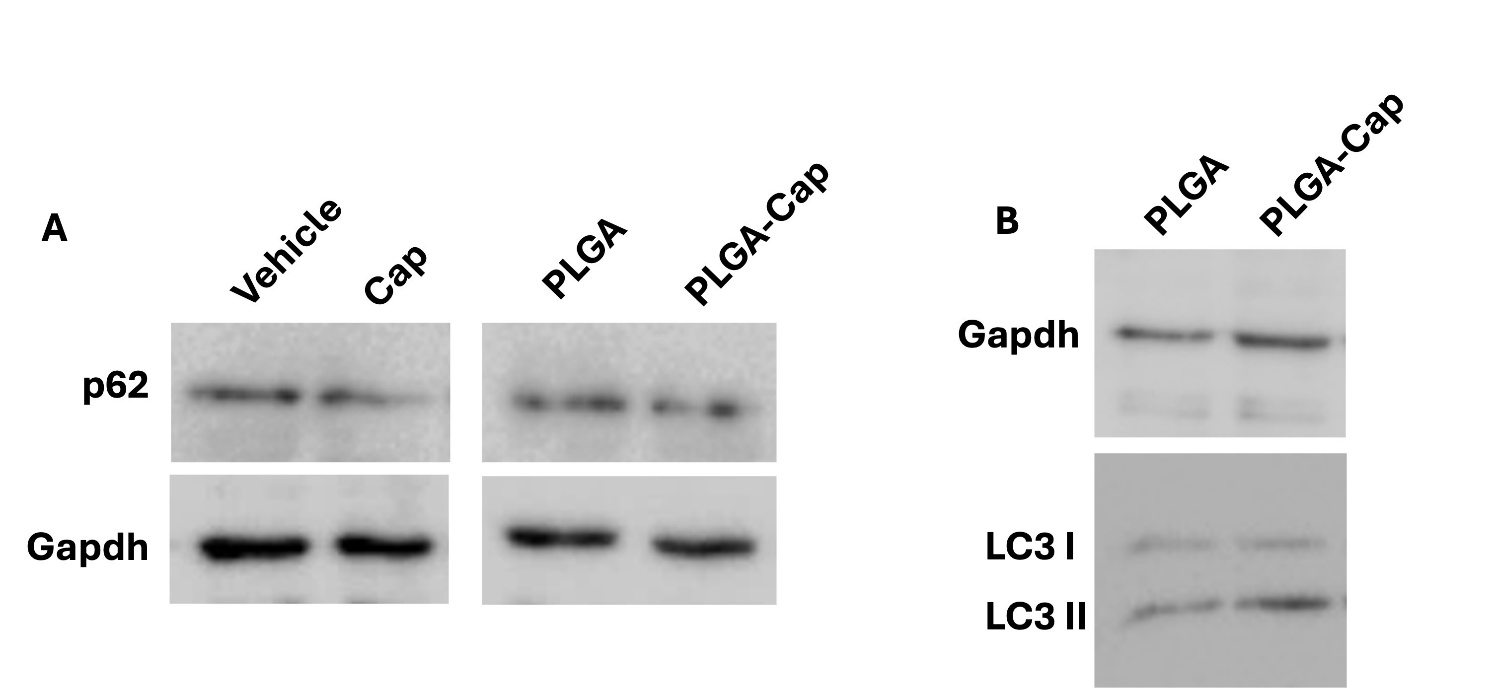


**Supplementary Figure 3.** Autophagy induction. (A) Western blot on 50 μg of proteins showing the level of p62 in cells treated for 24 h with 100 μM Cap and the respective vehicle (left), and with 100 μM of PLGA-Cap and PLGA (right). (B) Western blot on 50 μg of proteins showing the level of LC3 isoforms in cells treated for 24 h with PLGA-cap and PLGA.
